# Suppression of Inflammation and Arthritis by Orally Administrated Cardiotoxin from *Naja naja atra*

**DOI:** 10.1155/2015/387094

**Published:** 2015-02-12

**Authors:** Cao-Xin Chen, Jie-Yu Chen, Jian-Qun Kou, Yin-Li Xu, Shu-Zhi Wang, Qi Zhu, Lu Yang, Zheng-Hong Qin

**Affiliations:** Department of Pharmacology and Laboratory of Aging and Nervous Diseases, Jiangsu Key Laboratory of Translational Research and Therapy for Neuro-Psycho-Diseases, Soochow University School of Pharmaceutical Science, 199 Ren Ai Road, Suzhou 215123, China

## Abstract

Cardiotoxin (CTX) from *Naja naja atra* venom (NNAV) reportedly had analgesic effect in animal models but its role in inflammation and arthritis was unknown. In this study, we investigated the analgesic, anti-inflammatory, and antiarthritic actions of orally administered CTX-IV isolated from NNAV on rodent models of inflammation and adjuvant arthritis. CTX had significant anti-inflammatory effects in models of egg white induced nonspecific inflammation, filter paper induced rat granuloma formation, and capillary osmosis tests. CTX significantly reduced the swelling of paw induced by egg white, the inflammatory exudation, and the formation of granulomas. CTX reduced the swelling of paw, the AA clinical scores, and pathological alterations of joint. CTX significantly decreased the number of the CD4 T cells and inhibited the expression of relevant proinflammatory cytokines IL-17 and IL-6. CTX significantly inhibited the secretion of proinflammatory cytokine IL-6 and reduced the level of p-STAT3 in FLS. These results suggest that CTX inhibits inflammation and inflammatory pain and adjuvant-induced arthritis. CTX may be a novel therapeutic drug for treatment of arthritis.

## 1. Introduction

Rheumatoid arthritis (RA) is a systemic and progressive autoimmune disease characterized by joint inflammation, pain, cartilage, and bone destruction, which affects about 0.5–1% of population worldwide. The existing drugs to treat RA include nonsteroidal anti-inflammatory drugs, corticosteroids, disease-modifying antirheumatic drugs (DMARDs), and monoclonal antibodies [1]. But these drugs are often associated with various side effects and high cost [2]. Therefore, it is necessary to find new drugs for treatment of RA.

The natural product is a great treasure for medicine and biological drugs. Cobra venoms were used to treat pain, inflammation, and arthritis in traditional medicine of India and China. In recent years, various snake venom components have been used for drug development [3, 4]. A number of reports show that components from* Naja naja atra* venom (NNAV) have analgesic effects [5–8]. In addition, cobratoxin from Thailand cobra venom and Indian monocellate cobra venom was reported to have antiarthritic activity in several animal models [9, 10]. As a native protein drug, it is supposed that oral administration of snake venoms will be digested or degraded by various kinds of enzymes in gastrointestinal tract, just like other proteins. Deviation from our general idea, it was reported that oral administration of a low molecular weight component from* Crotalus durissus* venom and a neurotoxin from king cobra venom both produced analgesic effects [11, 12]. In addition, oral administration of denatured NNAV exerted analgesic and anti-inflammatory effects in arthritis models [13]. Cardiotoxin (CTX) is one of the most abundant components isolated from the NNAV; it produces analgesic effects in animal models of acute pain and neuropathic pain [14, 15]. Therefore, we speculate that oral administration of CTX might produce pharmacological effects in an adjuvant animal model of rheumatoid arthritis.

Recently, therapeutic strategies in RA had been shifted from controlling symptoms to potential restoring of immune system [16]. Immune system has an important impact in chronic inflammatory arthritis [17, 18]. T cells play a predominant role in the pathogenesis of RA, in particular CD4 T cells [19, 20]. Several relevant cytokines in RA have been reported to be involved in RA, including IL-1, IL-2, IL-6, IL-8, IL-10, IL-17, IL-23, TNF-*α*, and IFN-*γ* [17, 21–23]. IL-6 and IL-17 secreted by immune cells were reported to play crucial roles in pathogenesis of RA [23, 24]. Synoviocytes from RA patients or animal models morphologically composed of two cell types: macrophage-like synoviocytes (MLS) and fibroblast-like synoviocytes (FLS), whereby FLS play a crucial role in the initiation and pathogenesis of arthritis [25, 26]. To our knowledge, there is no report that has shown that orally administrated CTX produces anti-inflammatory effect in animal models and suppresses arthritis. In this study, we investigated if orally administrated CTX could produce analgesic, anti-inflammatory, and antiarthritic effects in animal models. We also investigated the influence of CTX on CD4 T cells and key proinflammatory cytokines (e.g., IL-6 and IL-17).

## 2. Materials and Methods

### 2.1. Animals and Reagents

The ethical committee for animal experiments at Soochow University School approved this study. Kunming mice and male Wistar rats were purchased from the Shanghai Slac Laboratory Animal Co., Ltd. Food and tap water were available adequately. Animals were maintained according to international guidelines for animal care. Cardiotoxin (CTX), purchased from Orientoxin Biotechnology Co., Limited (Lai-yang, Shandong Province, China), was dissolved in distilled water and was stored at −20°C until use. Complete Freund adjuvant (CFA) was purchased from Chondrex, Inc. (USA). The reasonable doses of the CTX were determined according to the LD50 of the CTX and preliminary test results in different animal models. All experimental research on animals in this study followed internationally recognized guidelines.

### 2.2. Analgesic Assay

To investigate the analgesic effect of CTX, we used acetic acid writhing assay and formalin test. Kunming mice (weighing 18–22 g) were used in these tests. CTX (5, 30, and 180 *μ*g/kg), the aspirin (100 mg/kg), and distilled water were orally pretreated for 5 days. In the acetic acid writhing assay, the number of writhes was counted from 5 min to 20 min after the injection of 0.1 mL/10 g 1.2% acetic acid saline solution to mice. In the formalin test, mice were subcutaneously injected 20 *μ*L 1.5% formaldehyde into the right hind paw. Then, time spent in licking the injected paw, divided into phase 1 (0–5 min) and phase 2 (15–30 min), was recorded [13, 27, 28].

### 2.3. Egg White Induced Nonspecific Inflammation Model

Rats were orally administrated with CTX (5, 30, and 180 *μ*g/kg) or Tripterygium wilfordii polyglycoside (TWP) (45 mg/kg) once daily for 5 days before injection of 0.1 mL of 10% (v/v) fresh egg white into the right hind paws. Paw volume was measured using the method of water displacement at 0, 0.5, 1, 2, 4, and 6 h after injection. The change in paw volume before and after injection of egg white was calculated.

### 2.4. Filter Paper Induced Rat Granuloma Formation Test

Filter papers were soaked in 7% formalin and then placed in the subcutaneous tissues at axillary area of the rats under sterile conditions. After surgery, rats were orally treated with CTX (5, 30, and 180 *μ*g/kg), TWP (45 mg/kg), or distilled water once daily for 7 days. All rats were killed and granulomas were taken out at 7th day (the most severe period of granuloma formation), and then the fresh granuloma was weighed as wet weight. After drying for 12 h at 60°C, the dry granuloma was weighed as dry weight.

### 2.5. Capillary Osmosis Test

KM mice were injected 0.5% Evans blue saline solution 0.1 mL/10 g by tail intravenous injection ten minutes after administration of 0.1 mL/10 g (i.p.) 0.6% acetic acid saline solution. Then 20 minutes later, the mice were killed and each abdominal cavity of the mouse was washed with 6 mL saline. The washed saline constant volume to 10 mL and centrifuged for 20 min at 4000 rpm/min, then read at 590 nm.

### 2.6. Induction and Evaluation of AA

Male Wistar rats (weighing 160–220 g) were used. Adjuvant arthritis (AA) was induced by intradermal injection of 0.1 mL complete Freund adjuvant (CFA) which contains 5 mg/mL heat-killed mycobacterium tuberculosis H37Ra (Mtb) into right paws. As a control, 0.1 mL of saline was injected. After the injection, these rats were observed periodically for swelling, erythema, and stiffness in each paw. The ankle joint circumference was measured using a flexible tape and the paw volume was measured with water displacement method.

To investigate the possible anti-inflammatory effect of orally treated CTX on the early days after CFA injection, CTX (45, 90, and 180 *μ*g/kg) was orally pretreatment once daily for 5 days before CFA injection. Paw volume and ankle joint circumference were measured at 0 (time before injection), 6, 24, and 72 h after CFA injection. To investigate whether orally treated CTX could suppress AA, rats were orally treated with CTX (45, 90, and 180 *μ*g/kg) from the 8th day after injection of CFA till the end of experiment. The paw swelling was measured by paw volume and ankle joint circumference at 0 (time before CFA injection), the 12th, 20th, 25th day after CFA injection.

In addition, AA was also induced by intradermal injection of 0.1 mL CFA at the base of tail. As evaluation of AA, swelling and erythema were scored on a scale of 0–4 for the severity of arthritis as arthritis score and the right ankle joints of rats were examined by histological assessment, respectively.

### 2.7. ELISA Assay

Blood was collected from abdominal aorta of each rat, respectively. After standing for 2 h, blood was centrifuged 3000 rpm for 15 min. Then the serum was collected and kept at −80°C for further analysis. The levels of IL-6, IL-10, and IL-17 were determined with the commercially available enzyme immunoassay kits (eBioscience, San Diego, CA, USA).

### 2.8. Blood Analysis and Flow Cytometric Analysis of T Cells

EDTA-anticoagulant peripheral blood was analyzed by automatic classification of five blood analyzer (CELL-DYN 3700, Abbott, Santa Clara, California, USA). The percentage of T lymphocyte subpopulations from 100 *μ*L EDTA-anticoagulant peripheral blood was determined with Flow cytometric analysis. After red blood cell lysis, the precipitated cells were washed with PBS once. Then the cells were stained with anti-rat CD3-PE, CD4-FITC, and CD8a-PEcy7 antibodies (eBioscience, USA) for 20 min in the dark at room temperature. Then cells were washed with PBS to remove excess stains. Each sample was suspended in 500 *μ*L of PBS and analyzed with a flow cytometer (FC500, Beckman Counter, USA).

### 2.9. FLS Culture and Treatment

The synovial fibroblasts were harvested from synovial tissues of AA rats at 16th day. Synovial fibroblasts were purified and authenticated with vimentin (+) (BioVision, USA) and CD68 (−) (Santa Cruz) for the tests. Purified synovial fibroblasts were restimulated* in vitro* with or without IL-1*β* (10 ng/mL) and treated with different concentrations of CTX for 6 h. Cell supernatant was collected for determination of the levels of IL-6. The lysates from these cells were prepared, and the levels of p-STAT3 were analyzed with immunoblotting.

### 2.10. Statistical Analysis

Calculations were performed using the SPSS 16.0 statistical package. One-way ANOVA with the Bonferroni posttest was carried out for* in vivo* experiments. All data were presented as mean ± SD. The post hoc test was Student's Newman Keuls test for quantitative values. *P* values less than 0.05 were considered as significant.

## 3. Results and Discussion

### 3.1. CTX Reduced Inflammatory Pain

**Figure 1:**
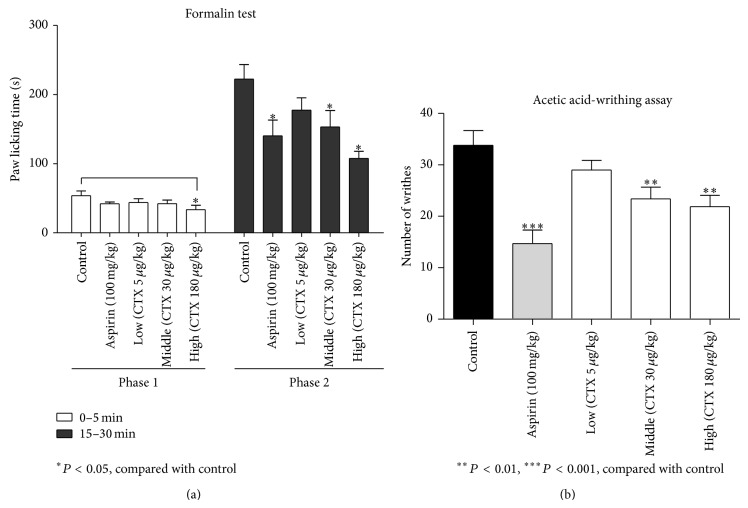
Attenuation of pain by orally administrated CTX. Mice were divided into five groups (*n* = 12 per group). CTX was orally administered for 5 days. In the Formalin test (a), time spent in licking the injected paw, divided into phase 1 (0–5 min) and phase 2 (15–30 min), was recorded; pretreatment with CTX (30, 180 *μ*g/kg) or aspirin all significantly inhibited the time spent in licking the injected paw compared with control in phase 2 (*P* < 0.05). CTX (180 *μ*g/kg) was more potent than aspirin (100 mg/kg). In the acetic acid writhing assay (b), the number of writhing movement was counted, pretreatment with CTX (30, 180 *μ*g/kg) or aspirin significantly reduced number of writhes. ^*^ *P* < 0.05, ^**^ *P* < 0.01, ^***^ *P* < 0.001, compared to control group.

We used the acetic acid writhing assay and the formalin test to investigate the analgesic effects of CTX. In the formalin test, pretreatment with CTX (30, 180 *μ*g/kg) or aspirin all significantly inhibited the time spent in licking the injected paw compared with control in phase 2 (*P* < 0.05, Figure 1(a)). CTX (180 *μ*g/kg) was more potent than aspirin (100 mg/kg). In the formalin test phase 1, aspirin or CTX (30 *μ*g/kg) did not significantly inhibit the acute pain response, but large dose of CTX (180 *μ*g/kg) did. In the acetic acid writhing assay, pretreatment with CTX (30, 180 *μ*g/kg) or aspirin significantly reduced number of writhes (Figure 1(b)). These results suggest that oral CTX has an analgesic effect. Since both formalin and acetic acid cause inflammatory pain, we speculated that CTX might have anti-inflammatory effects.

### 3.2. CTX Inhibited Inflammation in Animal Models

**Figure 2:**
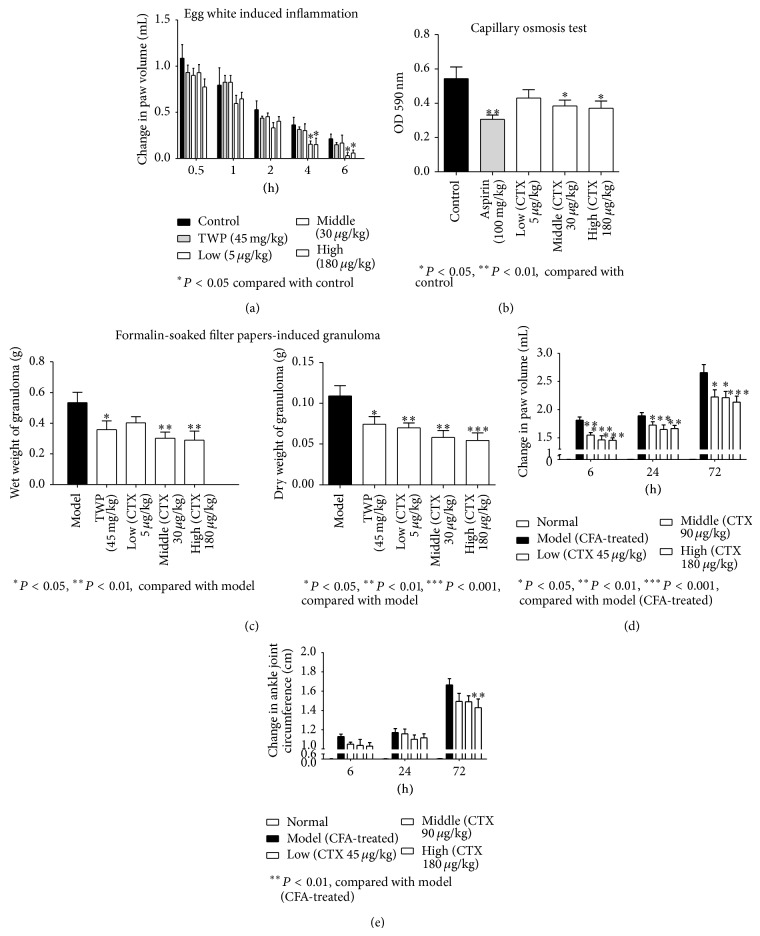
Attenuation of inflammation by orally administrated CTX. Wistar rats (*n* = 8 per group) were orally administrated with CTX (5, 30, and 180 *μ*g/kg), TWP (45 mg/kg), or distilled water. Paw volume was measured at 0, 0.5, 1, 2, and 4 h after injection of egg white. The change in swelling paw volume was measured (a); treatment with CTX (30, 180 *μ*g/kg) significantly reduced the swelling of paw at 4 and 6 h after egg white injection. In Capillary osmosis test (b), the injection of acetic acid solution into the abdominal cavity of mouse stimulated the capillary inflammatory exudation; this was reduced by aspirin or CTX (30, 180 *μ*g/kg). Granulomas in rats were induced by 7% formalin-soaked filter papers. Compared to the model group, the TWP and CTX (30, 180 *μ*g/kg) all alleviated the weight of granulomas, but CTX might be more efficacious (c). The CFA-induced primary inflammatory signs were recorded at 6, 24, and 72 h. The changes in paw volume (d) and change in ankle joint circumference (e) were shown. Pretreatment with CTX (45, 90, and 180 *μ*g/kg) for 5 days before the injection of CFA significantly reduced the swelling of paw at 6, 24, and 72 h, and pretreatment with CTX (180 *μ*g/kg) group significantly reduced ankle joint circumference at 72 h after CFA injection. ^*^ *P* < 0.05, ^**^ *P* < 0.01, ^***^ *P* < 0.001, compared to model group ((c), (d), and (e)) or control group ((a), (b)).

Egg white produced robust swelling of injected paw. The treatment with CTX (30, 180 *μ*g/kg) significantly reduced the swelling of paw at 4 and 6 h after egg white injection (Figure 2(a)). Consistent with some previous report, TWP did not produce significant anti-inflammatory effect [13, 29]. The injection of acetic acid solution into the abdominal cavity of mouse stimulated the capillary inflammatory exudation; this was reduced by aspirin or CTX (30, 180 *μ*g/kg) (Figure 2(b)). The implantation of the formalin-soaked filter paper caused the formation of granulomas. The granulomas were taken out at the 7th day after the modeling surgery, wet and dry weights of granulomas were measured. Compared to the model group, the TWP and CTX (30, 180 *μ*g/kg) all alleviated the weight of granulomas (Figure 2(c)), but CTX might be more efficacious. The injection of CFA induced acute inflammation of the injected paw in earlier period. Then, a general inflammation and symptoms appeared from the 10th day to 27th day after CFA injection. Pretreatment with CTX (45, 90, and 180 *μ*g/kg) for 5 days before the injection of CFA significantly reduced the swelling of paw at 6, 24, and 72 h (Figure 2(d)). Although pretreatment with CTX failed to reduce ankle joint circumference at 6 and 24 h, we found that pretreatment with CTX (180 *μ*g/kg) group significantly reduced ankle joint circumference at 72 h after CFA injection (Figure 2(e)). These results suggest that orally administered CTX suppresses inflammation.

### 3.3. CTX Suppressed Adjuvant Arthritis

**Figure 3:**
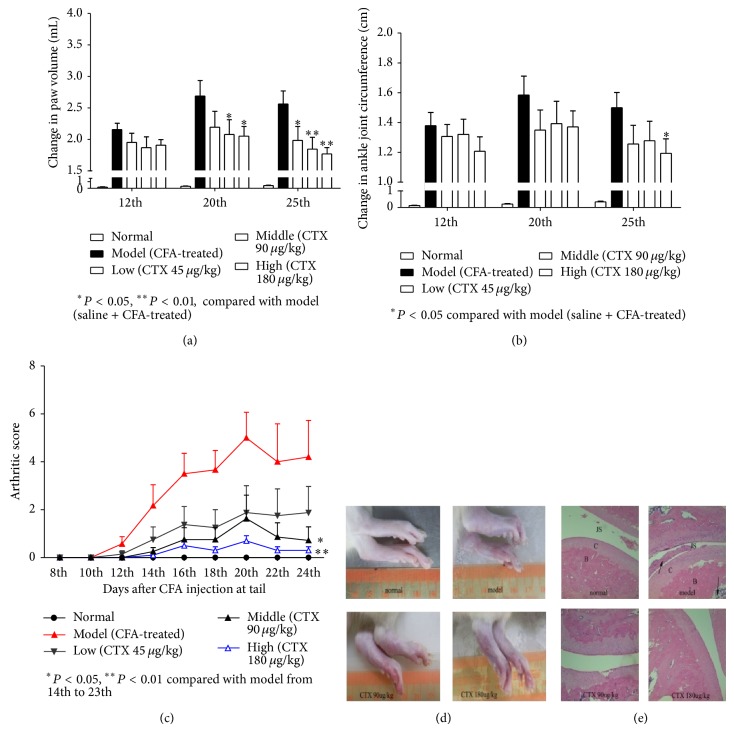
Suppression of AA in Wistar rats by orally administrated CTX. Wistar rats (*n* = 8 per group) were orally administrated with CTX (45, 90, and 180 *μ*g/kg) or distilled water. AA was induced by intradermal injection of 0.1 mL CFA into the right paw, and the changes in paw volume and ankle joint circumference before and after CFA injection were determined at 12th, 20th, and 25th. There was no significant difference in paw volume and ankle joint circumference among the groups at the 12th day, while the paw swelling was significantly alleviated in CTX (90, 180 *μ*g/kg) treated groups at 20th day and 25th day (a). CTX (180 *μ*g/kg) treated group also significantly reduced ankle joint circumference at 25th day (b). AA induced by intradermal injection of 0.1 mL CFA at the base of tail was measured on a clinical scale of 0–4 for the severity of arthritis (c). Pictures of the hind paws of a representative Wistar rat from each group at 25th day after injection of CFA at the base of tail are shown (d). The sections of HE stained hind paws (e). In picture (e), B: bone; C: cartilage; JS: joint space and the arrows indicate infiltration of inflammatory cells. ^*^ *P* < 0.05, ^**^ *P* < 0.01, ^***^ *P* < 0.001, compared to model group.

We determined antiarthritic effect of CTX in rat adjuvant arthritis (AA) model. There are usually four different phases in a typical course of AA: incubation, onset, summit, and recovery. Because of the general inflammation and symptoms that appear from the 10th day after CFA injection, we orally administrated drugs once daily from 8th day to investigate the therapeutic effects of CTX. We measured paw volume and ankle joint circumference to evaluate the inflammation and symptoms of AA at 0th, 12th, 20th, 25th day after CFA injection. We found that there was no significant difference in paw volume and ankle joint circumference among the groups at the 12th day, while the paw swelling was significantly alleviated in CTX (90, 180 *μ*g/kg) treated groups at 20th day and 25th day (Figure 3(a)). CTX (180 *μ*g/kg) treated group also significantly reduced ankle joint circumference at 25th day (Figure 3(b)). We also induced AA by intradermal injection of 0.1 mL CFA at the base of tail and use the arthritic score to evaluate the pathological condition. The mean arthritic scores of CTX (180 *μ*g/kg) treated and control rats at the summit course of the disease were 0.7 and 5.0, respectively (Figure 3(c)). Compared to the model group, oral administration of CTX displayed a significantly lower mean arthritic score (Figure 3(c)). The antiarthritic activity of orally treated CTX was further confirmed by histological assessment of the hind paws (Figure 3(e)). Joint space, synovial inflammatory cell infiltration, cartilage, and bone destruction were all significantly ameliorated in the joints of CTX treated rats compared with the model rats. These results showed that oral CTX effectively suppressed adjuvant arthritis.

### 3.4. CTX Reduced Serum IL-6 and IL-17 in AA Rats

**Figure 4:**
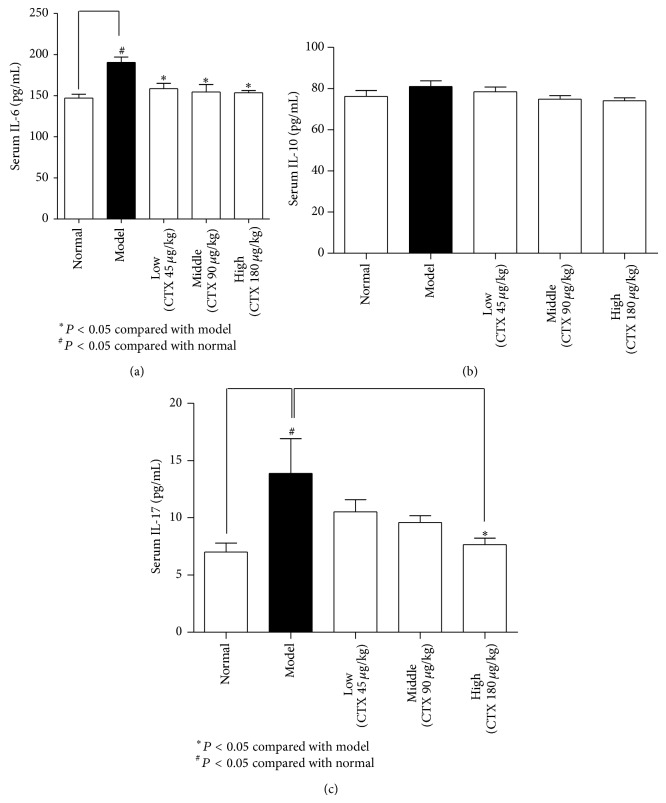
CTX reduced serum IL-6 and IL-17 in AA rats. Blood serum was collected at 25th day after CFA injection. Serum levels of IL-6, IL-10, and IL-17 were determined with enzyme immunoassay kits ((a), (b), and (c)). The level of IL-6 in serum of AA model rats was significantly higher than that in normal rats, while the levels of IL-6 in CTX (45, 90, and 180 *μ*g/kg) treated AA rats were significantly lower than that in model rats (a). Similar to the IL-6 expression, the levels of IL-17 in serum of AA model rats were significantly higher than that in normal rats, which was significantly attenuated by CTX (180 *μ*g/kg) (c). However, there was no difference in IL-10 level among normal and AA rats with or without CTX administration (b). Values are the mean ± SD of 8 rats per group. ^*^ *P* < 0.05, compared with model group; ^#^ *P* < 0.05, compared to normal group.

**Figure 5:**
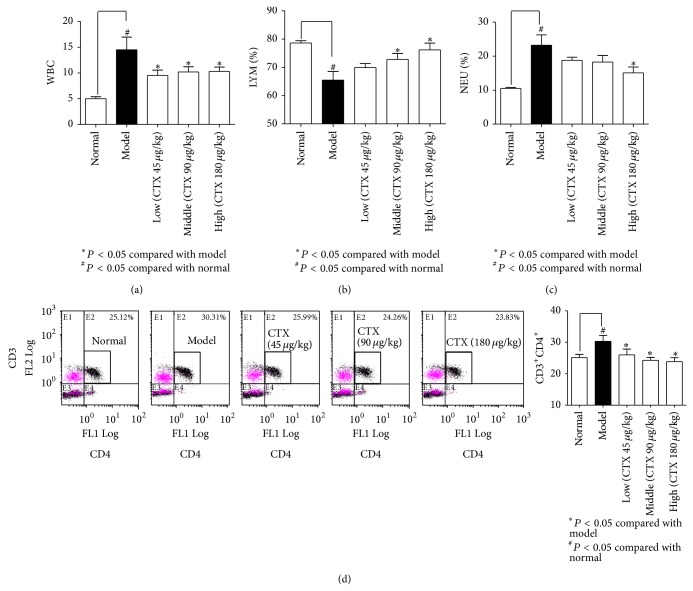
The effects of CTX on peripheral blood cells in AA rats. EDTA-anticoagulant peripheral blood was analyzed by automatic classification of five blood analyzer. The number of white blood cells (WBC), lymphocytes (LYM) ratio, and neutrophil cell (NEU) ratio were measured ((a), (b), and (c)). CTX reduced WBC number in AA rats (a), restored lymphocyte number, and reversed the elevation of neutrophil cells ((b) and (c)). Flow cytometric analysis of T lymphocyte subpopulations, CD3^+^CD4^+^ T cell ratio, is shown in picture. There was a significant increase in the number of CD4^+^ T cells in AA rats compared with normal rats, and CTX significantly decreased CD4^+^ T cells in AA rats (d). ^*^ *P* < 0.05, compared with model group; ^#^ *P* < 0.05, compared to normal group.

The level of IL-6 in serum of AA model rats was significantly higher than that in normal rats, while the levels of IL-6 in CTX (45, 90, 180 *μ*g/kg) treated AA rats were significantly lower than that in model rats (Figure 4(a)). Similar to the IL-6 expression, the levels of IL-17 in serum of AA model rats were significantly higher than that in normal rats, which was significantly attenuated by CTX (180 *μ*g/kg) (Figure 4(c)). However, there was no difference in IL-10 level among normal and AA rats with or without CTX administration (Figure 4(b)). These results revealed that CTX inhibited the expression of critical cytokines related to arthritis. EDTA-anticoagulant peripheral blood of experimental animals was analyzed by automatic classification of five blood analyzer. CTX reduced white blood cell (WBC) number in AA rats (Figure 5(a)), restored lymphocyte number, and reversed the elevation of neutrophil cells (Figures 5(b) and 5(c)). Flow cytometric analysis of T lymphocyte subpopulations in peripheral blood indicated that there was a significant increase in the number of CD4^+^ T cells in AA rats compared with normal rats, and CTX significantly decreased CD4^+^ T cells in AA rats (Figure 5(d)). Reports have shown that immune system plays a major role in chronic inflammatory arthritis [17] and T cells play a predominant role in the pathogenesis of RA, in particular CD4^+^ T cells [19, 20, 30]. These results showed that orally administrated CTX could suppress T cell-mediated immune response.

### 3.5. CTX Modulated Cytokine-Related Transcription Factors in FLS

**Figure 6:**
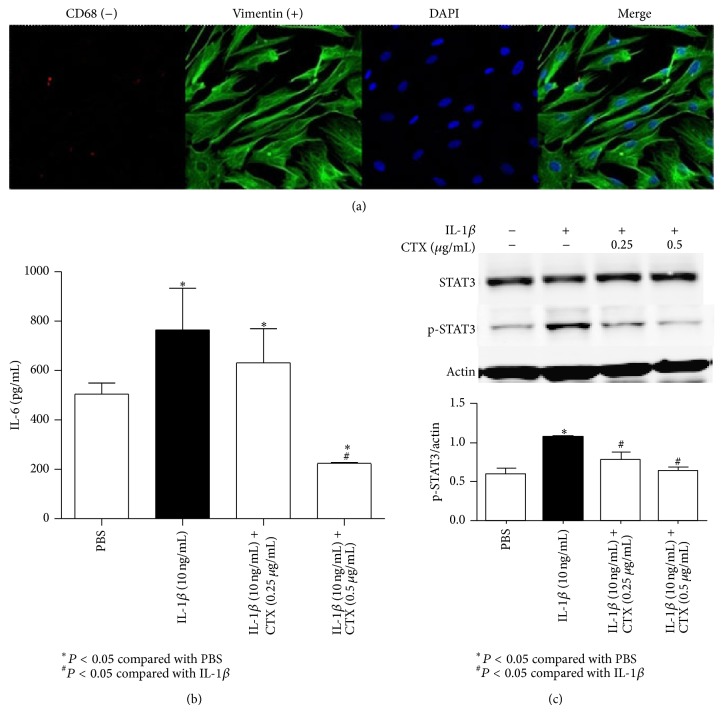
CTX modulated cytokine-related transcription factors in synovial fibroblasts. The synovial fibroblasts were harvested from synovial tissues of AA rats at 16th day. Synovial fibroblasts were purified and authenticated with vimentin (+) and CD68 (−) for the tests (a). Purified synovial fibroblasts were restimulated* in vitro* with or without IL-1*β* and treated with CTX for 6 h. Cell supernatant collected for determination of IL-6 (b); the levels of IL-6 were enhanced when stimulated with IL-1*β*, whereas it dropped down when given CTX (0.25, 0.5 *μ*g/mL) at the same time. Lysates from these cells were prepared, and the levels of p-STAT3 were analyzed with Western blotting (c). The levels of p-STAT3 were increased when stimulated with IL-1*β*; it dropped down when given CTX (0.25, 0.5 *μ*g/mL) at the same time. ^*^ *P* < 0.05, compared with PBS group; ^#^ *P* < 0.05, compared with IL-1*β* group.

The fibroblast-like synoviocytes (FLS) were harvested from synovial tissues of AA rats at 16th day of CFA injection. Macrophage-like synoviocytes (MLS) were marked with CD68 and fibroblast-like synoviocytes were marked with vimentin. Synovial fibroblasts were purified and authenticated with vimentin (+) and CD68 (−) for the tests (Figure 6(a)). Purified synoviocytes were almost more than 90% fibroblast-like synoviocytes in the present study. FLS were stimulated* in vitro* with or without IL-1*β* and treated with CTX for 6 h. Cell supernatant was collected and IL-6 in cell supernatant was measured (Figure 6(b)); the levels of IL-6 were enhanced when stimulated with IL-1*β*, whereas it dropped down when given CTX (0.25, 0.5 *μ*g/mL) at the same time. Lysates from these cells were prepared, and the levels of p-STAT3 were analyzed with Western blotting (Figure 6(c)). The levels of p-STAT3 were increased when stimulated with IL-1*β*; it dropped down when given CTX (0.25, 0.5 *μ*g/mL) at the same time.

## 4. Discussion

The major finding of this study is that orally administrated CTX produced a therapeutic effect in CFA arthritis. This notion was supported by the positive results of CTX on inflammation induced by egg white, acetate, and filter papers; on inflammatory pain response induced by acetic acid and formalin; and on joint swelling induced by CFA and the clinical manifestation and joint pathology induced by systemic injection of CFA. Aspirin, a classical anti-inflammatory drug, was used as positive control in inflammatory animal models. We found that CTX provides a slightly better anti-inflammatory effect in acetic acid and formalin tests. Tripterygium wilfordii polyglycoside (TWP) is a traditional Chinese medicine used for treatment of rheumatoid arthritis in clinic. It was reported that TWP had immunosuppressive and anti-inflammatory effects in the history of clinical use [31, 32]. Nonetheless, reports showed that low dosage of TWP did not produce significant anti-inflammatory effect [13, 29], and the use of TWP often produced adverse effects in clinical therapy, such as anorexia, diarrhea, and abdominal pain. In the present study, TWP displayed anti-inflammatory effect in filter paper induced rat granuloma formation test; however, we found that its effect was less potent than that of CTX.

It has been reported that cobra venom and components from NNAV were used to treat pain, inflammation, and arthritis [5–8, 12–14, 33]. In addition, cobra venom factor (CVF) effectively inhibited arthritis in animal models as well [34–36]. Studies showed that CTX had analgesic effects, antitumor property, and potential bactericidal activity [14, 33, 37, 38]. Researches of CTX provide its activities of heart toxicity and muscle contraction [39, 40]. To our knowledge, the toxicity of CTX is much lower than neurotoxin from NNAV and NNAV. Cobra venom has been used for treatment of pain and herpes viral infection in USA for many years. Several allergic reactions have never been reported [41]. In our study, orally administrated CTX induced no toxicity and allergic reaction at rational dose in murine animals. However, no studies had focused on the anti-inflammation and antiarthritic effects of CTX. CTX is belonging to the super family of neurotoxin and is the most abundant component in NNAV. As far as we know, CTX was reported to be stable in artificial gastric juice and mouse's stomach. No smaller fragments were detected* in vivo* and* in vitro* assays [42]. We tentatively believe that it was intact CTX delivery to the local inflammatory sites for the anti-inflammatory activities at the present. We have obtained experimental evidence that oral administration of NNAV or neurotoxin can produce pharmacological effects [5, 8, 13]. Since oral administration is convenient and feasible, we investigated the anti-inflammatory and antiarthritic effects of orally administrated CTX in animal models. Interestingly, oral administration of CTX produced anti-inflammatory effect in filter paper-induced granuloma formation in rats and acetic acid-induced abdominal capillary permeability. Meanwhile, CTX suppressed CFA-induced primary inflammation. These results suggest that orally administrated CTX could produce anti-inflammatory effects.

Subsequently, our studies showed that oral administration of CTX displayed potent antiarthritic effects in a rat AA model. CTX significantly reduced CFA-induced ankle swelling and joint inflammatory damage. Interestingly, orally administrated CTX affected cellular immune system, significantly declined the number of CD4^+^ T cells in AA rats. T cells play an important role in autoimmune disease and inflammatory response [43, 44]. A number of basic and clinical studies found that CD4 T cells play an important role in the pathogenesis of RA [20, 45, 46]. Based on the importance of CD4 T cells in arthritic disease and inflammatory response, it is deserved to determine the influence of CTX on CD4 T cells. Our present research revealed that CTX inhibited CD4 T cells. At the same time, we did not see a significant difference of CD8^+^ T cells among each group. The role of CD8 T cells in the pathogenesis of arthritis is controversial; report shows that CD8 T cells in the pathogenesis of arthritis are not essential in HLA-B27 transgenic Rats [47].

Several relevant cytokines have been reported to play a role in the initiation and progression of RA, including IL-1, IL-2, IL-6, IL-8, IL-10, IL-23, TNF-*α*, and IFN-*γ* [17, 21, 22]. Recently, a number of studies paid close attention to the “Th17” T helper cell and its cytokine IL-17 in the progression of RA [18, 48]. Th17 cell is a crucial subpopulation of CD4 T cells, and IL-17 is a proinflammatory cytokine secreted by Th17 cells. It is proposed as a future therapeutic target for RA by some investigators [23, 49–51]. IL-6 is also a crucial cytokine involved in arthritic [24, 52]. IL-6 induces the differentiation of Th17 cells from naive precursors [53]. From our study, CTX significantly inhibited the expression of IL-17 and IL-6 but had no effect on the anti-inflammatory cytokine IL-10. The results suggest that CTX might selectively restrain Th17 cells or inhibit the IL-6 to recede the differentiation of Th17 cells from naive precursors. Our latest research demonstrated that NNAV affected cellular immune system and could selectively inhibit CD4 Th17 [54], lending the support that CTX has a role in regulating Th17 cells. In brief, these results showed that orally administrated CTX suppressed inflammatory reaction and arthritis via regulation of cellular immune system.

To further elucidate the molecular base of an antiarthritis effect of CTX, we studied the inhibitory effects of CTX on synoviocytes. Fibroblast-like synoviocytes (FLS) are key cells that display both passive responders and aggressors in the process of RA [55, 56]. STAT3 is an essential transcription factor in Th17 differentiation, activation, proliferation, and survival [57]. As a key transcription factor in Th17 differentiation, activation, proliferation, and survival, it is becoming a relevant treatment target for RA and other autoimmune diseases. Inhibition of the key transcriptional factors, p-STAT3, was correlation with the decrease of Th17 cells in autoimmune arthritis model [58, 59]. The JAK2/STAT3 cascade is responsible for IL-6-mediated cellular responses in both physiological and pathological events. The interference with STAT3 signalling could be a good therapeutic strategy to mitigate autoimmune diseases, including RA [60]. In our study, CTX reduced the IL-1*β*-induced elevation of IL-6 and p-STAT3 protein levels in FLS. These factors are involved in differentiation and function of Th17 cells, thus assumed to play a regulatory role in AA. The influence of CTX on IL-6 and p-STAT3 in FLS may help us to understand the pharmacological effects of CTX on AA.

## 5. Conclusion

Oral administration of CTX exerted analgesic, anti-inflammatory, and antiarthritic actions. Orally administrated CTX attenuated the manifestation of AA and inhibited the CD4 T cells. Finally, CTX significantly inhibited the expression of relevant proinflammatory cytokines, including IL-17 and IL-6. Comparing to some other antiarthritic drugs, CTX may have certain advantages owing to its analgesic, anti-inflammatory, and immunoregulation effects. We believe that this study will provide new avenue for developing novel therapeutic strategies for arthritis.

## Abbreviations

CTX: Cardiotoxin
NNAV: * Naja naja atra* venom
RA: Rheumatoid arthritis
AA: Adjuvant arthritis
STAT3: Signal transducer and activator of transcription 3
TWP: Tripterygium wilfordii polyglycoside
CFA: Complete Freund adjuvant
FLS: Fibroblast-like synoviocytes
MLS: Macrophage-like synoviocytes
Mtb: Heat-killed mycobacterium tuberculosis H37Ra
IL: Interleukin
WBC: White blood cell
PBS: Phosphate-buffered saline
Th: CD4-positive helper T cells
TNF-*α*: Tumor necrosis factor alpha.
